# Supplementary material for: The transcriptomic response to heat stress of a jujube (Ziziphus jujuba Mill.) cultivar is featured with changed expression of long noncoding RNAs
Source: PLoS One. 2021 May 27;16(5):e0249663. doi: 10.1371/journal.pone.0249663 (PMC8158912; doi:10.1371/journal.pone.0249663)
Supplement: Attachment — Submitted filename: comments for plos one.docx [file pone.0249663.s011.docx]

Comments:

The manuscript which is named “The transcriptomic response to heat stress of a jujube (Z*iziphus jujuba* Mill.) cultivar is featured with changed expression of long noncoding RNAs” analyzed the

lncRNAs which might play an important role in response to HS in Chinese jujube. However, some modifications need to be done to improve the manuscript.

The most important question for this manuscript is the language. I strongly suggest the language of this manuscript should be polished by native speaker. For example, in the abstract, what is “not also, but also”. Introduction, “biotic stressors”、 “is involved response to”“was made to

be”and so on. Discussion “Our found a putative cultivar (Hqing1-HR) and bred it in our laboratory” and so on, these sentences are difficult to understand.

Then the character of the cultivar Hqing1-HR should be described in the introduction part, why chose this material to do heat stress?

The results parts are superficial, after reading I do not get any interesting information. Also the discussion part should be rewrote and discuss more lncRNAs in Chinese jujube.The authors should explain the results and give the reasons.
