# Supplementary material for: The transcriptomic response to heat stress of a jujube (Ziziphus jujuba Mill.) cultivar is featured with changed expression of long noncoding RNAs
Source: PLoS One. 2021 May 27;16(5):e0249663. doi: 10.1371/journal.pone.0249663 (PMC8158912; doi:10.1371/journal.pone.0249663)
Supplement: Attachment — Submitted filename: A rebutal letter.docx [file pone.0249663.s012.docx]

Dear editors,

Thank you for arranging a timely review for our manuscript (Manuscript ID: PONE-D-20-29127). We have carefully evaluated your critical comments and thoughtful suggestions, and improved the manuscript accordingly. We are glad to resubmit the MS right now.

The relevant comments and our responses are presented below.

We look forward to hearing from you soon, and will further improve the MS according to your kind suggestions.

Best regards!

Qing Hao

**Reviewer 1#**

Hao et al, perform RNA -seq profiling of the jujube plant following heat stress. They identify differentially expressed lncRNAs over a time course of heat stress. They investigate the possibility that some lncRNAs are correlated with their nehioring mRNAs and could be involved in cis regulation. They confirm the induction of a small number of lncRNAs identified in the RNA-seq by qPCR. This study provides a resource on genes dysregulated during heat stress in jujube. I have some comments below that if addressed will make this publication acceptable for publication in Plos one.

**Major：**

**Comment 1:** The authors say 51% (4632) were long intergenic noncoding RNAs (lincRNAs), how are they defining intergenic. Is there a threshold for how far away the gene must be from a neighboring protein coding gene? (to ensure it is not simply an unnotated start site).

**Response 1:** Thanks. We were sorry for our negligence. In fact, there is a threshold that lincRNAs must be more than 2000 bp away from the neighboring protein coding genes. We provided this information in the resubmitted MS.

**Comment 2:** When the authors say “In our previous study, we also explored the 2266, 4907, 6120 and 2894 DEGs in HR1 VS HR0, HR3 VS HR0, HR5 VS HR0 and HR7 VS HR0, respectively (Data not shown).” Is this a published study? If so please add the reference instead of data not shown. Overall this entire section as written is very confusing to me. Are the authors looking at the cis acting effects of the differentially expressed lncRNAs from table 1 or from another study entirely? I think it is from this study but as it is written right now it is very confusing.
**Response 2:** We were sorry for the confusing and mistaken written English. Actually, we obtained RNA-seq data presented in our previous study, and explored the differentially expressed (DE) lncRNAs (S2 Table), DE mRNAs (S4 Table), cis-target mRNAs of DElncRNAs (S5, S6 Tables) in the current study. We wrote this section and provided the supplementary information (S4-S6) in the resubmitted MS.

**Comment 3:** The entire section on “DE lncRNAs regulate the metabolic process”. I am not sure how informative this section is to simply list out all of the metabolic processes. I don’t think outlining each one in necessary. This whole section could be summarized into a couple of sentences.

**Response 3:** Thanks. This whole section had been summarized into a couple of sentences in the resubmitted MS.

**Comment 4:** Figure 2C. It would be useful to the reader to add the common 40 lncRNAs that are differentially expressed into a separate table.

**Response 4:** Thanks for your good suggestion. A table (**S3 Table**) containing these 40 DE lncRNAs was presented in the resubmitted MS.

**Comment 5:** The authors say “To our surprise, it was found that 1911, 3474, 4066 and 1996 cis-acting target mRNAs were overlapped with DEGs in HR1 VS HR0, HR3VS HR0, HR5 VS HR0 and HR7 VS HR0 (Fig 3 A, C, E and G)”. What do they mean as targets? I think it would be very useful to break it down into the mRNAs that show the same pattern of induction as the neighboring lncRNA and which are anticorrelated in expression level (where the mRNA is up and the lncRNA is down or vice versa). Placing this lists of potential cis regulators together within a table would be a good resource to accompany Figure 3.
**Response 5:** Thanks a lot. We rewrote these sentences in this section. Moreover, A table (**S6 Table**) containing the lists of potential cis regulators was presented in the resubmitted MS.

**Comment 6:** The authors say “Moreover, the results of qRT-PCR experiments were consistent with the results from RNA-seq (Fig. 4A) with Pearson correlation coefficients (PCCs) higher than 0.9, suggesting the accuracy of these data”. This data comparison is now shown. It would be useful to show the data and what they are measuring the PCCs within this figure.

**Response 6:** Thanks for good suggestion. A “Fig.4C” measuring the PCCs was added in the resubmitted MS.

**Minor:**
**Comment 7:** Define DE first before abbreviating
Esthetically it is nice to have font similar sizes between graphs within the same figure

**Response 7:** Thanks. We have adjusted the abbreviating and the font sizes between graphs within the same figure in the resubmitted MS.

**Reviewer #2**

The manuscript which is named “The transcriptomic response to heat stress of a jujube (Ziziphus jujuba Mill.) cultivar is featured with changed expression of long noncoding RNAs” analyzed the lncRNAs which might play an important role in response to HS in Chinese jujube. However, some modifications need to be done to improve the manuscript.
**Comment 1:** The most important question for this manuscript is the language. I strongly suggest the language of this manuscript should be polished by native speaker. For example, in the abstract, what is “not also, but also”. Introduction, “biotic stressors”, “is involved response to”“was made to be”and so on. Discussion “Our found a putative cultivar (Hqing1-HR) and bred it in our laboratory” and so on, these sentences are difficult to understand.
**Response 1:** Thanks. We have tried our best to improve the language in the resubmitted MS. We would like to improve it further, according to your kind suggestions.

**Comment 2:** Then the character of the cultivar Hqing1-HR should be described in the introduction part, why chose this material to do heat stress?

**Response 2:** Thanks for kind suggestion. We added the corresponding information in the introduction part.

**Comment 3:** The results parts are superficial, after reading I do not get any interesting information. Also the discussion part should be rewrote and discuss more lncRNAs in Chinese jujube. The authors should explain the results and give the reasons.

**Response 3:** Thanks. We rewrote multiple sentences, added some information of lncRNAs in the results and discussion parts. We will continue to improve the MS according to your kind suggestions.
